# Task-Involving Motivational Climate and Enjoyment in Youth Male Football Athletes: The Mediation Role of Self-Determined Motivation

**DOI:** 10.3390/ijerph20043044

**Published:** 2023-02-09

**Authors:** Nuno Amaro, Diogo Monteiro, Filipe Rodrigues, Rui Matos, Miguel Jacinto, Beatriz Cavaco, Sandro Jorge, Raúl Antunes

**Affiliations:** 1 ESECS—Polytechnic of Leiria, 2411-901 Leiria, Portugal; 2 Life Quality Research Centre (CIEQV), 2400-901 Leiria, Portugal; 3 Research Center in Sport, Health, and Human Development (CIDESD), 5000-558 Vila Real, Portugal; 4 Center for Innovative Care and Health Technology (ciTechCare), 2415-396 Leiria, Portugal

**Keywords:** football, coaches, achievement goal theory, self-determination theory

## Abstract

Background: This study aimed at examining the mediation role of self-determined motivation (identified and integrated regulation and intrinsic motivation) in the association between task-involving climate and enjoyment in youth male football athletes. Methods: A total of 109 youth males (M = 14.38; SD = 1.55) were recruited to participate in this study. The survey included sociodemographic data and validated instruments such as the Motivational Climate Sport Youth Scale, the Behavioral Regulation Sport Questionnaire, and the Sports Enjoyment Scale. Results: The results showed that the task-involving climate was a positive and significant predictor of integrated regulation and intrinsic motivation. In addition, integrated regulation and intrinsic motivation were positive and significant predictors of enjoyment. The results of the mediation analysis revealed a partial mediation role of self-determined motivation in the relationship between task-involving climate and enjoyment. Significant indirect effects only occurred through intrinsic motivation. Conclusions: Providing higher levels of enjoyment in the sport context could be an excellent option for leisure activities for children and youth, as long as self-determined motivation and task-involving climates provided by the coaches are present.

## 1. Introduction

Youth participation in sports represents a healthy way to provide physical, psychological, and social benefits [1,2]. Therefore, factors that could explain sports participation, particularly in youths, represent a central topic for educational and political decision-makers and stakeholders such as teachers, educators, and coaches [3]. One of the biggest research challenges is the understanding of which factors can contribute to sports participation [4] and their influence on several physical, social, emotional, and cognitive outcomes in youth [5].

Several studies [6,7] have proposed that motivation is a nuclear variable that can transform physically inactive to active behaviors based on social, environmental, and interpersonal aspects [8]. The most comprehensive and contemporary theoretical frameworks for describing behavioral, cognitive, and emotional patterns in the sport context are the Self-Determination Theory [9] and the Achievement Goal Theory [10]. The Self-Determination Theory considers intrinsic motivation to be the prototype of self-determined behavior, indicating that the individual participates voluntarily in any give behavior (e.g., sports) and without any sort of reward or external pressure. This type of motivation is also integrally linked to the feelings of pleasure and fun associated with the behavior [6,9]. The Achievement Goal Theory is founded on motivational and achievement-related consequences in relation to how individuals perceive or define success, as a result of their interactions with goal-oriented contexts.

The relationships between the Achievement Goal Theory and Self-Determination Theory frameworks are documented in the literature [11,12,13]. These theoretical frameworks have been integrated to create a multi-theoretical framework of sport behavior [11] by examining how the context grounded in the Achievement Goal Theory assumptions influences motivation based on the Self-Determination Theory approach. Specifically, the Achievement Goal Theory suggests that the social context influences motivational regulation, with consequences on the behavior itself [14]. Therefore, the motivational climate created by sport coaches seems to influence self-determined motivation, since it may promote or hinder sport adherence [15]. It means that the social environment may provide the necessary conditions for self-determined motivation because controlling climates (e.g., an ego-involving climate) are typically associated with non-self-determined motivation, whereas climates that support competence (e.g., a task-involving climate) are typically associated with self-determined motivation [3]. Overall, the context may contribute to non-self-determined motivation if regarded as controlling and encouraging an ego-involving atmosphere, or it may lead to self-determined motivation if competence and personal development are supported [11].

### 1.1. Task-Involving Motivational Climate

The impact of significant others’ social support (e.g., coaches) on athletes’ motivation is documented in the literature [15], and results demonstrate that an adequate social support provided by sport coaches promotes a greater self-determined motivation [1]. Athletes’ motivation in sports may be influenced by coaches’ behavior [16]. Coaches design training sessions, and the interactions with players throughout training or competition can lead to improved intrinsic motivation [11] through the motivating climate that the coaches can create [17]. The motivational climate experienced by the athletes is related to the environment established by the coach during training and competitions, which is influenced by what they do in those situations and has an impact on how athletes think, feel, and act [10,17]. As a result, two types of climates emerge. One of them is the task-involving climate, which emphasizes learning and personal development and in which effort is rewarded and mistakes are considered part of the learning process [11]; the other one is the ego-involving climate, which emphasizes the competition between athletes, the demonstration of one’s competence when compared to others’, and in which the results are rewarded and the mistakes are punished. According to research on the motivational climate established by coaches and its effects on athletes of various competitive levels, when the climate is seen as task-involving, as opposed to ego-involving, the athletes tend to adopt more adaptive tactics that are beneficial to sport participation and are able to maintain their practice on the long term [18,19].

### 1.2. Self-Determined Motivation

The Self-Determination Theory [9] is a macro theory of human motivation that differentiates several types of motivation based on their degree of autonomy or self-determined behavior. This macro theory consists of six micro theories, each of which was designed to describe a set of motivationally driven phenomena [20]. We will consider the organismic integration theory which describes the motivational continuum of self-determined behavior and how the quality of motivation can influence emotional, cognitive, and behavioral aspects in athletes. Several pieces of data, notably, in the context of sport, have allowed grouping the six regulations into two macro dimensions called self-determined motivation and non-self-determined motivation [21,22]. Self-determined motivation assumes that a behavior is performed due to the positive values inherent in the behavior, while the person integrates the behavior into his/her daily lives, and includes identified regulation, integrated regulation, and intrinsic motivation, the three more self-determined forms of motivation present in the motivational continuum. Contrarily, non-self-determined motivation assumes that the performance of a behavior is adjacent to coercive or self-imposed assumptions and includes introjected regulation, external regulation, and in some instances, amotivation, the three least forms of self-determined behavior found in the motivational continuum. Individuals who operate on the basis of a regulated incentive are thus less likely to be self-determined in the long run [9].

### 1.3. The Role of Enjoyment in Youth Sport Participation

According to Ryan and Deci [9] and empirical evidence [21,22], sports motivation research has revealed that self-determined motivation, particularly intrinsic motivation, plays a crucial role in sport. In an ideal world, when athletes’ participation in sports is genuinely driven and affected by intrinsic motivation such as pleasure, it results in beneficial outcomes such as enjoyment [23]. In addition, it could mean that intrinsic motivation may be one of the most important factors in maintaining sports participation over time [3,6]. According to the self-determination theory assumptions, higher levels of self-determined motivation are associated with high levels of enjoyment, whereas higher levels of non-self-determined motivation are associated with lower levels of enjoyment [18,19,24].

Enjoyment has been examined to some extent in the context of sport [23]. It is one of the most important factors associated with sport initiation and maintenance, acting as a result of a task-involving motivational climate as well as a predictor of intentions towards future sport practice [6]. More specifically, enjoyment has been described as a multidimensional construct composed of positive activation, competence, attitude, and cognition [21,25]. In the sport context, enjoyment is a favorable response to the experience of performing sport [25] that is manifested by feelings of pleasure and flow. Furthermore, enjoyment is a positive emotional response that takes into account the athlete’s sports experience [26], which has been linked to both the intent to continue practicing and sport adherence [6].

### 1.4. The Role of Enjoyment in Youth Sport Participation

Although some studies have investigated the relationship between task-involving climate and self-determined motivation [27], as well as the associations between self-determined motivation and enjoyment [24,28], existing studies that address the mediation role of each type of self-determined motivation in relation to task-involving climate and enjoyment are limited [24]. The current study intends to fill this gaps, because each type of self-determined motivation may contribute in different ways [29] to predicting enjoyment and, as a result, could help coaches better tailor their interventions to promote enjoyment, which is one of the most important predictors of sport adherence [30]. Therefore, the aim of the present study was to analyze the mediation role of identified and integrated regulation and intrinsic motivation in the relationship between task-involving climate and enjoyment in youth male football athletes. We hypothesized that all forms of self-determined motivation would display a mediation role in the relationship between task-involving climate and enjoyment, since self-determined regulations are consequences of positive contextual factors [9,10] and enjoyment predicted by the degree of self-determined motivation [24]. Intrinsic motivation should show the greatest mediation role in the relationship between task-involving climate and enjoyment, since intrinsic motivation is the most significant predictor of enjoyment compared to identified and integrated regulation [10].

## 2. Materials and Methods

### 2.1. Participants

On hundred nine (n = 109) youth male football athletes were recruited by using the convenient sampling method to take part in the study. Eligible participants aged between 12 and 18 years (*M* = 14.38; *SD* = 1.55) were considered, and all athletes reported to practice football in a local club. The frequency of the practice was 2–4 times per week (*M* = 3.24; *SD* = 0.54).

### 2.2. Measures

The task-involving motivational climate was assessed using the Motivational Climate Sport Youth Scale [31], Portuguese version [7]. This scale comprises eight items, and the responses are evaluated on a five-point Likert scale, which fluctuates between 1 (“Totally Disagree”) and 5 (“Totally Agree”). The items are grouped into two factors (four items each) that reflect the theoretical constructs of the Achievement Goal Theory [10], namely, task-involving climate (e.g., “The coach encouraged us to learn new skills”) and ego-involving climate (e.g., “Coach told us to try to be better than our teammates”). In this study, only the task-involving climate subscale items were used and showed good levels of internal consistency (*α* = 0.80).

The Behavioral Regulation Sport Questionnaire [32], Portuguese version [33], to measure self-determined forms of motivation was used. A total of 24 items are part of this questionnaire, and the responses are evaluated on a seven-point Likert scale, which varies between 1 (“Nothing True for Me”) and 7 (“Totally True for Me”). The items are grouped into six factors (four items each) that reflect the types of motivation underlying the motivational continuum, namely, amotivation (e.g., “but I question why I continue”), external regulation (e.g., “to satisfy people who want me to play”); introjected regulation (e.g., “because I would feel guilty if I quit”); identified regulation (e.g., “because I value the benefits of my sport”), integrated regulation (e.g., “because it’s a part of who I am”) and intrinsic motivation (e.g., “because I enjoy it”). For the present study, identified and integrated regulation and intrinsic motivation subscale items were considered. The subscales showed good levels of internal consistency (identified regulation: α= 0.74; integrated regulation: α = 0.82; intrinsic motivation: *α* = 0.88).

The Physical Activity Enjoyment Scale [34], Portuguese version [35], was used to measure the cognitive aspect of enjoyment during sport practice. This questionnaire consists of eight items, and the responses are evaluated on a 5-point Likert scale ranging from 1 (“totally disagree”) to 5 (“totally agree”). The items are grouped into a single factor that reflects the level of enjoyment of the participants (e.g., “I find it pleasurable”), which is defined as a positive response to the experience of sports practice [25]. In the present study, this scale showed a good level of internal consistency (*α* = 0.89).

### 2.3. Procedures

Given that Leiria was chosen as the European City of Sport—2022 (LECS—2022)—a study of the local community’s sport participation was carried out. As a result, during an activity devised in this area, we queried a group of respondents about their sports-related practice. A total of 121 young people were interviewed. Of these, 115 said that they participated in sport by playing football in local clubs. As a result, the local clubs were called in order to gain the necessary authorization to continue this study. After receiving a positive response to continue the investigation, the children’s parents or legal tutors were contacted. During this encounter, the nature, ethical, and data collection procedures of the study were presented. Afterwards, the parents or legal tutors provided an informed consent granting permission for their children to participate in this research. Six potential participants missed the data collection date on the day the surveys were administered and where thus not included in this study. As a result, 109 young male athletes were included in the final sample. On the day of the questionnaire distribution, all information on the nature of this inquiry was delivered to the male athletes. They were also told that they might opt out of the study at any moment. The subjects gave their agreement before completing the questionnaire, and anonymity was maintained. All data collection procedures were in accordance with the Helsinki Declaration [36]. The data were collected at the beginning of the training session, and the time required for the completion of the questionnaires was approximately 15 min. Before the data collection, ethical approval was obtained from the Polytechnic of Leiria Ethics Committee (PARECER N.º CE/IPLEIRIA/24/2021).

### 2.4. Statistical Analysis

We used the expectation–maximization approach to handle possible missing completely at random data. Means, standard deviations, and bivariate correlations were computed for the variables under consideration. To determine the statistical significance of deviations from the normal distribution, the skewness and kurtosis estimates were divided by their corresponding standard error to obtain the z score. Z scores below |1.96| suggest a normal distribution. The significance level was set at *p* ≤ 0.05 for the correlations between the variables of interest. These analyses were conducted in IBM SPSS Statistics version 26.0 (IBM Corp., Armonk, NY, USA).

Mediation methods were used according to Hayes [37]. IBM PROCESS version 3.5 macro was used to conduct the mediation analysis. Task-involving motivational climate, all forms of self-determined motivation, and enjoyment were employed in the model as manifest variables, computed by calculating the mean of the scale items. Based on the mediation analysis assumptions [37], a mediation model was tested (model 4, with three parallel mediators). Specifically, predictor variable (i.e., task-involving), outcome variable (i.e., enjoyment), and three parallel mediators (i.e., identified, integrated regulation, and intrinsic motivation) were imputed in the mediation model for the analysis. It is worth mentioning that this is a sequential model, and thus, the variables were inserted in the model according to previously cited theoretical and empirical evidence. This approach estimates the direct and indirect effects in the given models while controlling for the role of k mediators between variables (Hayes, 2018). Bias-correct bootstrapped estimates (with standard errors and 95% CI) were computed for the dependent–independent variables interaction. Significant indirect effects were considered if the confidence interval did not include zero (at alfa = 0.05). For this study, a 5000-sample bootstrapping, bias-corrected, and confidence intervals were considered. As suggested by Hayes [37] and MacKinnon et al. [38], bootstrapping procedures are recommended since they are more efficient and powerful than the normal theory approach for detecting indirect effects in smaller samples.

Before performing the mediation analysis, a tolerance test was performed and Variance Inflation Factor (VIF) scores were analyzed to test for possible multicollinearity issues [39]. The tolerance test of independent variables should be greater than 0.1, and the VIF should be less than 10 for no detection of possible multicollinearity issues. The results showed that the VIF and tolerance tests scores were below 10 and above 0.1, respectively, ensuring the appropriate conditions to test the mediation model.

## 3. Results

### 3.1. Preliminary Analysis

A preliminary examination discovered no missing values or outliers. The mean scores for intrinsic motivation were greater compared to those for the other forms of self-determined motivation. The skewness and kurtosis values were below the cutoff values, indicating a normal distribution. Several significant bivariate correlations emerged as expected: (a) a task-involving climate was positively and significantly associated with integrated regulation and intrinsic motivation; (b) all forms of self-determined regulation were positively and significantly associated with enjoyment (for details see Table 1).

Before performing the mediation analysis, the tolerance test and Variance Inflation Factor (VIF) scores were analyzed to test for possible multicollinearity issues [39]. The tolerance of independent variables should be greater than 0.1 for there to be no multicollinearity. The collinearity diagnosis was checked via the variance inflation factor (VIF) and the tolerance test, assuming values less than to 10 for VIF and greater than to 0.01 for the tolerance test. Therefore, the results showed that the VIF and the tolerance tests scores were below 10 and above 0.1, respectively, ensuring the appropriate conditions to test the mediation analysis.

### 3.2. Mediation Analysis

**Figure 1:**
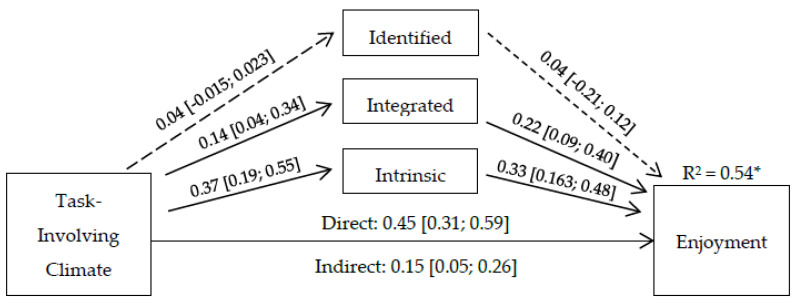
Multiple mediation analysis. Note: * *p* < 0.001, between squared brackets = confidence interval at 95%, full arrow = significant effect, dashed arrow = non-significant effect.

Figure 1 displays the results of the mediation model. A partial mediation was identified, since both total direct and indirect effect were significant. However, the significant indirect effects only occurred through intrinsic motivation (*β* = 0.12 [0.04–0.21]). The indirect paths between task-involving climate and enjoyment via integrated (*β* = 0.01 [−0.02–0.01] and identified regulation (*β* = 0.03 [−0.01–0.11]) were not significant. In total, the explained variance of the model for enjoyment was 54% (*p* < 0.001).

## 4. Discussion

Grounded on the Achievement Goal Theory and Self-Determination Theory assumptions, the goal of this study was to examine the mediation role of self-determined motivation (identified and integrated regulation and intrinsic motivation) in the relationship between task-involving climate and enjoyment in youth male football athletes. Overall, the findings indicated a partial mediation, since both total direct and indirect effects were significant. The results will be discussed considering the existing literature.

The current findings revealed that young male football athletes value all constructs here considered, since all analyzed variables displayed means higher than the mid-point. These findings are consistent with earlier research [24,40,41], which indicated the significance of task-involving motivational climate, self-determined motivation, and enjoyment for football practice. The correlations pattern revealed positive and significant correlations across the studied variables, except for task-involving motivational climate and identified regulation. This evidence is supported by previous studies that were conducted with these theoretical models in athletes involved in several sports, such as swimming [42], football [40], rugby [43], as well as other individual and team sports [41]. While competence-supportive climates promote self-determined motivation, this may be only true for the motivational regulations that consider sport as an integrated part of ones ‘self. Thus, the lack of statistical significance for the association between task-involving climate and identified regulation could be due to the nature of the identified regulation subscale. It is important to note that the mentioned studies [40,41,42,43] examined self-determined motivation as a composite factor, which means that the association between task-involving climate and identified regulation could not be confirmed, and the literature on this topic appears to be scarce. Moreno-Murcia et al. [44] discovered a positive relationship between ego-involving motivational climate and identified regulation in a sample of 413 athletes aged 12 to 16 years, explaining that external pressures could influence young athletes in identifying sport practice as valuable. In addition, as previously highlighted by Villadrich et al. [45], the absence of discriminant validity between identified and integrated regulation in the measurement of behavioral regulations in young athletes could potentially account for this finding [33,46].

According to the results of the mediation analysis, task-involving motivational climate displayed a positive and significant association with integrated regulation and intrinsic motivation. In turn, these behavioral regulations are positive and significant predictors of enjoyment in the context of football practice. These associations are supported by theoretical and empirical evidence [11,27,30]. More specifically, the Achievement Goal Theory theorizes key aspects of motivation at the contextual level, since this model suggests that contextual factors such as interpersonal behaviors provided by coaches that are less evaluative and more supportive to the intrinsic desire to learn may promote improvements in achieving the desired competence and goals of adolescents [11]. In fact, the task-involving motivational climate and its attributes, in which learning and personal progress are accepted, effort is rewarded, and errors are acknowledged as a normal part of the education process, are very similar to sports pedagogy approaches commonly used in youth training [47]. Furthermore, during adolescence, there are pubertal fluctuations, incipient ability to think about oneself, changes in different roles and responsibilities, as well as personality development. Thus, the motivational climate created by the coach could be understood as a crucial factor that functions to normalize and increase well-being and enjoyment throughout these adolescent years and act as a promoter of sport practice [29]. Therefore, our results are in line with those of prior studies that showed that athletes who perceive that their coaches create a task-involving motivational climate exhibit more self-determined motivational patterns [27] and thus, consequently, a greater perception of enjoyment [23].

Several research studies reported similar results when it comes to the analysis of the association between self-determined motivation and enjoyment. For example, Álvarez et al. [23] found a positive and significant relationship between self-determined motivation and enjoyment (*β* = 0.33), as well as a negative and significant relationship between self-determined motivation and boredom (*β* = −0.22) among Spanish football athletes. This finding is also consistent with the self-determination theory assumptions, which suggests that when athletes report being more self-determined, they are more likely to report higher levels of adaptive emotional consequences such as positive affect and enjoyment [20]. Since intrinsic motivation can be conceptually defined (and expressed in behavioral terms) as the enjoyment and pleasure that people feel when performing a particular behavior, it is theoretically possible to assume that young athletes that experience greater pleasure are able to have more fun and experience positive affect when playing football.

The mediation model demonstrated partial mediation because both direct and indirect effects were significant. The strong indirect effects occurred mainly because of intrinsic motivation. Grounded on the Self-Determination Theory assumptions, the results could be explained according to theoretical evidence, since intrinsic desire is one of the most important variables on long-term sport adherence [9,24]. This leads us to believe that the processes regulating youth emotional reactions, particularly in this age range, are tied to intrinsic drive. In a Spanish sample of young athletes, Álvarez et al. [23] found this tendency by demonstrating that intrinsic motivation partially mediates the connection between autonomy-supportive behaviors provided by sport coaches and enjoyment. Furthermore, Ruiz et al. [27] showed that self-determined motivation was a partial mediator in the relationship between task-involving motivational climate and the influence of functional anger in a sample of Finnish athletes.

The non-significant indirect effect of identified and integrated regulation in the association between task-involving climate and enjoyment could be due to the extrinsic nature of these motivational regulations. While identified regulation refers to the level of personal investment of an individual in an activity, young football athletes could see the motivational climate as reinforcing only the intrinsic side of motivation, Thus, when these male athletes engage in football for the purpose of meeting their own goals and needs, they are said to have high levels of identified regulation, meaning that enjoyment could be at risk of not being met (as seen in Figure 1). Related to integrated regulation, while it refers to an individual’s internalization of the values, goals, and interests linked to an activity, it is still an extrinsic form of motivation. Hence, while self-determined by nature, in young male athletes who perceive their coaches as task-involved, it does not translate to integrating football as personally important. However, a task-involving climate explains perceived enjoyment directly and indirectly via intrinsic motivation, showing the importance of self-determined behavior for positive emotional outcomes.

### Limitations and Agenda for Future Research

Although the current study helps to understand the role of each form of self-determined motivation in the relationship between task-involving climate and enjoyment in youth male football athletes, it has some limitations. Because all variables were evaluated at the same time (i.e., cross-sectional design), we could only discuss the connections between variables without determining causality. In this regard, longitudinal or experimental research are required to further investigate the effects of the variables we considered. In addition, although statistically significant, the sample size was small and comprised only male football athletes. Therefore, future studies should make an effort to examine the respective associations between the studied variables in larger samples, in relation to other sport modalities, and in female athletes. It also appears vital that characteristics such as competitive level and experience be incorporated in future studies.

This study makes an essential contribution to this research topic and leaves some indicators for sport coaches to consider. On the one hand, it is critical that coaches manage to improve task-oriented cultures, which appear to be related to higher levels of pleasure and enjoyment and, as a result, a larger likelihood of athletes remaining in practice. On the other hand, coaches must understand how their athletes manage their motivation and can also boost higher levels of self-determined motivation, specifically, intrinsic motivation (e.g., with the inclusion of self-referenced criteria in the task components). Athletes who regulate their behavior autonomously have more pleasant practice outcomes, and our study appears to highlight the mediation role of self-determined motivation (particularly, intrinsic motivation) in the association between task-involving motivational climate and enjoyment.

## 5. Conclusions

Among the various ways to occupy male children and adolescents with sports, football, in this case, appears to bring a set of biopsychosocial benefits that reinforce the importance of health-related behaviors during the adolescent years as well as the need to implement technical and political strategies to increase the levels of sport practice. The main finding is that a partial mediation role of self-determined motivation in the relationship between task-involving climate and enjoyment was observed. Specifically, the strong indirect effects occurred mainly because of intrinsic motivation. We believe that this study contributes that can assist athletes’ coaches, as well as sport, educational, and policy stakeholders in developing efficient interventions for promoting higher levels of enjoyment. A sport context that encourages higher levels of enjoyment could be a good strategy to foster football participation during a person’s lifespan.

## Figures and Tables

**Table 1:** Range, means, standard deviation, and bivariate correlation across the studied variables.

Variables	Range	M	SD	1	2	3	4	5
1. Task-involving climate	3–5	4.49	0.41	1	-	-	-	-
2. Identified Regulation	2–7	5.02	1.06	0.04	1	-	-	-
3. Integrated Regulation	1–7	5.58	1.14	0.15 *	0.59 **	1	-	-
4. Intrinsic Motivation	4–7	6.40	0.69	0.37 **	0.16 *	0.46 **	1	-
5. Enjoyment	3–5	4.09	0.70	0.60 **	0.15 *	0.41 **	0.5 8 **	1

Note. M = Means; SD = Standard deviation; * = *p* < 0.05; ** = *p* < 0.001.

## Data Availability

The data were used under license exclusively for the current study. The data that support the findings of this study are not publicly available but are available upon reasonable request and with permission of the Life Quality Research Center and the corresponding author.
